# Occurrence of species of the genus *Pityophthorus* Eichhoff (Coleoptera, Curculionidae, Scolytinae) in the province of Quebec, Canada

**DOI:** 10.3897/zookeys.348.6029

**Published:** 2013-11-12

**Authors:** Valentin Popa, Louis Morneau, Céline Piché, André Deshaies, Eric Bauce, Claude Guertin

**Affiliations:** 1 INRS-Institut Armand-Frappier, 531 des Prairies Boulevard, Laval, Quebec, Canada, H7V 1B7; 2 Ministère des Ressources naturelles du Québec, DPF, 2700 Einstein Street, Québec, Quebec, Canada, G1P 3W8; 3 Ministère des Ressources naturelles du Québec, DGPSP, 880 Chemin Sainte-Foy, Québec, Quebec, Canada, G1S 4X4; 4 Université Laval, DBF, 2320 Des Bibliothèques Street, Québec, Quebec, Canada, G1V 0A6

**Keywords:** Distribution, fauna, locality, Quebec, records, twig beetle

## Abstract

Twig beetles in the genus *Pityophthorus* Eichhoff, 1864 include more than 300 species worldwide, with maximum diversity in tropical and subtropical regions. To date, approximately 50 species of *Pityophthorus* have been recorded in Canada, and these species are associated mainly with coniferous trees. Since 1981, no comprehensive study on this difficult taxonomic group has been conducted in Quebec, Canada, most likely due to their limited significance as forest pests. Based on data gathered from five years of field sampling in conifer seed orchards and compiled from various entomological collections, the distribution of *Pityophthorus* species in Quebec is presented. Approximately 291 new localities were recorded for the *Pityophthorus* species. Five species-group taxa, namely *Pityophthorus puberulus* (LeConte, 1868), *Pityophthorus pulchellus pulchellus* Eichhoff, 1869, *Pityophthorus pulicarius* (Zimmermann, 1868), *Pityophthorus nitidus* Swaine, 1917,and *Pityophthorus cariniceps* LeConte&Horn, 1876 were the most widespread. In contrast, *Pityophthorus consimilis* LeConte, 1878, *Pityophthorus intextus* Swaine, 1917, *Pityophthorus dentifrons* Blackman, 1922, *Pityophthorus ramiperda* Swaine, 1917, and *Pityophthorus concavus* Blackman, 1928 display a notably limited distribution. In addition, the first distribution records of *Pityophthorus intextus* and *Pityophthorus biovalis* Blackman, 1922 are furnished, and the subspecies *Pityophthorus murrayanae murrayanae* Blackman, 1922is reported from Quebec for the second time. Moreover, distribution maps are provided for all *Pityophthorus* species recorded in the province of Quebec.

## Introduction

Twig beetles in the genus *Pityophthorus* Eichhoff, 1864 include approximately 386 species distributed worldwide (Bright 1981; Bright and Skidmore 1997, 2002; Wood and Bright 1992) with 218 species found in North and Central America (Bright 1981; Wood 1982), and approximately 55 other species found in South America (Wood 2007). More than 70 % of the *Pityophthorus* world fauna is distributed in North, Central, and South America. Wood (2007) notes that the genus *Pityophthorus* has an American origin and displays its maximum diversity in subtropical and tropical areas. A vast majority of the North and Central American species of this genus breed in twigs of coniferous trees (Bright 1981; Wood 1982), whereas the South American species breed predominantly in deciduous trees (Wood 2007).

Excluding the taxonomic works, twig beetles have received little attention from the scientific community and forest managers simply because these organisms generally colonize declining trees or tree parts. They are often found in thin-barked parts of stressed or weakened trees, and they are rarely associated with extensive epidemics in forest ecosystems (Furniss and Carolin 1977). Nevertheless, some reports have indicated that several *Pityophthorus* species may become problematic and cause medium to severe damage in coniferous plantations (Rappaport and Wood 1994; Stevens et al. 1979). Furthermore, twig beetles may vector pathogenic fungi. For example, pitch canker disease of Monterey pine, *Pinus radiata* D. Don, is caused by wounding and transmission of a fungal pathogen to twigs during feeding by a complex of *Pityophthorus* sp. in California (Dallara 1997; Hoover et al. 1996; Sakamoto et al. 2007). In a Monterey pine plantation in Spain, approximately 25 % of the analyzed population of *Pityophthorus pubescens* (Marsham) was responsible for carrying the spores of *Fusarium circinatum* Nirenberg and O’Donnell, which is the fungus that causes pitch canker disease (Romón et al. 2007). More recently, Kolařik et al. (2011) reported that another *Pityophthorus* species, the walnut twig beetle, *Pityophthorus juglandis* Blackman, is an important vector of the fungus *Geosmithia morbida* (M. Kolařik, E. Freeland, C. Utley and Tisserat 2011). This complex causes thousand cankers disease, which is a serious necrosis of the phloem of walnut trees, *Juglans* sp., in the United States (Seybold et al. 2013). The walnut twig beetle has expanded its range considerably in the U.S. (Cranshaw 2011; Seybold et al. 2012), which may explain the recent attention that the disease has attracted from the forest management community (Seybold et al. 2013).

The species of the genus *Pityophthorus* are difficult to identify with morphological techniques. Both sexes are often required for accurate identification at the species level (Bright 1981; Wood 1982, 2007). Taxonomic revisions of this genusin North America have been published by LeConte and Horn (1876), Swaine (1918), and Blackman (1928). Wood (1978) placed the genus *Pityophthorus* into the Corthylini and the Pityophthorina (subtribe). The *Pityophthorus* are closely related to *Araptus* Eichhoff, 1872 whose species are found mainly in Mexico, Central, and South America (Wood 2007; Wood and Bright 1987, 1992). A major contribution to the knowledge of the *Pityophthorus* was provided by Bright (1981), who published a taxonomic monograph in which more than 220 species from Central and North America were described and classified.

In Canada, approximately 50 species of *Pityophthorus* have been recorded (Bright 1968; Bright 1971, 1976, 1981; Bright and Skidmore 1991, 1997, 2002; Bright et al. 1994; Wood and Bright 1987, 1992). In the province of Quebec, Canada, 17 species have been reported (Bright 1976, 1981; Bright and Skidmore 1997, 2002; Laplante et al. 1991; McNamara 1991; MRNQ 2008). However, no comprehensive and up-to-date study has been performed on the genus *Pityophthorus* in Quebec since the publications of D.E. Bright.

The purpose of this article is to update the distribution of all recorded *Pityophthorus* species in the province of Quebec, Canada. The species records are based on data collected from several entomological collections and on our own field-trapping data obtained during the last five years (2008–2012). A study of the distribution of *Pityophthorus* species is highly important, particularly in ecological and biological studies on different species and for further taxonomic revisions of this difficult bark beetle group. This study also provides background information for researchers working with exotic forest insects and for forest managers.

## Methods and conventions

This article is based on data obtained from *Pityophthorus* specimens collected exclusively in the province of Quebec, Canada. Two different sources of data were used: field captures performed between 2008 and 2012 and a survey of public and private entomological collections.

*Field collection methods*. The field data included in this article were collected over a period of five years (2008–2012) from trapping activities conducted in six different seed orchards scattered from west to east over diverse types of landscapes in the province of Quebec (Table 1). These seed orchards are composed mainly of white pine, *Pinus strobus* L., but also contain specimens of red pine, *Pinus resinosa* Ait. and jack pine, *Pinus banksiana* Lamb., as well as white spruce, *Picea glauca* (Moench) Voss, red spruce, *Picea rubens* Sargent, and black spruce, *Picea mariana* Miller, Briton, Sterns & Poggenburg.

**Table 1.** The sampled seed orchards with their corresponding area in hectares, their geographic coordinates and the nearest locality.

**Seed orchard**	**Nearest locality**	**Geographic coordinates (decimal degrees)**	**Area (ha)**
Huddersfield	Fort-Coulonge	45.9215; -76.6219	9.7
Dorion	Lac Cayamant	46.0459; -76.2828	6.6
Verchères	Saint-Amable	45.6773; -73.3303	5.5
Cleveland	Saint-Claude	45.6764; -71.9954	2.5
Aubin-de-l’Isle	Saint-Simon-les-Mines	46.2089; -70.6780	4.5
Cap-Tourmente	Cap-Tourmente	47.0680; -70.8076	2.8

Two types of trapping techniques were used in the study sites. The first trapping technique employed “Yellow Japanese Beetle” (YJB) traps (Trécé Inc., Adair, Oklahoma, United States) equipped with 500-ml Mason® jars. Each jar was filled with 50 ml of propylene glycol to kill and preserve the trapped insects. The YJB traps were baited with a polyethylene “bubble cap” release device (Contech Enterprises Inc., Delta, British Columbia, Canada) that contained (±) *trans*-pityol (release rate of 0.2 mg/day). Pityol is an aggregation pheromone component of several *Pityophthorus* species (Brauner and De Groot 2007; Dallara et al. 2000; De Groot and De Barr 2000; Francke et al. 1987; López et al. 2011). The population monitoring of *Pityophthorus* species in the six seed orchards was performed starting in 2008 and ending in 2012. The biological material was collected with an average frequency of twice a month from mid-April to the end of September.

A second trapping technique was used to increase the chance of capture of other *Pityophthorus* specimens that may not respond to synthetic pityol alone as an attractant. Twelve-unit Lindgren funnel traps were used to monitor *Pityophthorus* populations in three of the six previously mentioned seed orchards, namely Verchères, Huddersfield and Cleveland (Table 1). The Lindgren traps were placed at a density of approximately three traps per hectare and were baited with (±) *trans*-pityol and UHR ethanol (200 mg/day, Synergy Semiochemicals Corp., Burnaby, British Columbia). Ethanol is an attractant for large number of bark and ambrosia beetles (Miller and Rabaglia 2009). Lindgren traps were equipped with plastic trap cups filled with 50 ml of propylene glycol. The cups were emptied at the same frequency as described in the previous trapping technique. The field-collected *Pityophthorus* specimens were preserved in 70  % ethanol and subsequently mounted and pinned. All of the captured specimens originating from field trapping are preserved in the INRS-Institute Armand-Frappier (INRS-IAF) entomological collection. The species identification was performed according to morphological criteria by using a Discovery V-20 stereomicroscope (Carl Zeiss Canada Ltd.) equipped with an ICc3 video camera. Pictures of the identified and unidentified *Pityophthorus* species are available at the following web address: www.profs.inrs.ca/cguertin/ZOOKEYS_2013/MENU.html

The field data were complemented with information gathered from six public and two private entomological collections. In this article, the following acronyms are used for the public entomological collections:

**CNC** Canadian National Collection of Insects, Arachnids and Nematodes, Agriculture and Agri-Food Canada, Ottawa, ON, Canada;

**MRNQ** Ministère des Ressources Naturelles du Québec, Québec, QC, Canada;

**LFRC** Natural Resources Canada, Laurentian Forestry Research Center, René-Martineau Insectarium, Québec, QC, Canada;

**ECLU** Entomological Collection, Laval University, Québec, QC, Canada;

**LEMU** Lyman Entomological Collection, McGill University, Montreal, Quebec, Canada;

**ROUM **Robert-Ouellet Entomological Collection, Montreal University, Montreal, QC, Canada.

The acronyms used for the private entomological collections are the following:

**CCC** Claude Chantal’s Collection, Varennes, QC, Canada

**CLC** Claire Lévesque’s Collection, Sherbrooke, QC, Canada

The following two additional acronyms are also employed in this article:

**SLWC** S.L. Wood entomological collection. Although this collection was not directly examined, some *Pityophthorus lautus* Eichhoff specimens captured in the province of Quebec are deposited there. Even though the specimens have not been examined, these are included in the article as reliable records that were published by Bright (1981).

**INRS**-**IAF **Entomological collection of the INRS-Institut Armand-Frappier, Laval, QC, Canada. All specimens collected during the field trapping activities are deposited in this collection.

Approximately 50 % of the specimens analyzed in this study belong to MRNQ entomological collection. In general, to capture bark beetle specimens, the “Ministère des Ressources naturelles du Québec” (MRNQ) uses permanent sampling stations dispersed in all representative types of forest ecosystems and landscapes across the province of Quebec.

*Other conventions and symbols*. The distribution record for each of the *Pityophthorus* species mentioned in this article is displayed using the following sequences: 1. Name of the locality where the specimen was captured. In some cases, instead of the name of the locality, the historic territorial administrative name may appear (e.g., “Township”), because no locality name has been assigned to the sampling area. 2. Name of the Regional County Municipality (RCM) to which the cited locality belongs. Regional County Municipalities have delineated the province of Quebec territory since 1979. Many localities in Quebec that are situated in different geographic areas have received the same name. To avoid any confusion relative to the locality names, the corresponding RCM is included. 3. Date of capture. If the capture date is missing or it is unreadable on the label, a question mark is included. 4. Number of examined specimens and the acronym of the entomological collection to which they belong. These data are included within parentheses and separated by a coma. 5. Name of host tree species, displayed in italics. In some cases, the host tree data are missing.

The five abovementioned sequences are separated by comas. Locality records are separated by semicolons (;). If a species was recorded many times in the same locality, the various dates of capture are separated by a slash symbol ( / ). For each mentioned species, the distribution data are presented in a manner that previous literature records are separated from the new records to highlight the originality of the article. The general distribution of each species in Canada is presented at the end of each species record. The following abbreviations were used for the provinces and territories: **YT**-Yukon Territory, **NT**-Northwest Territories, **NU**-Nunavut, **BC**-British Columbia, **AB**-Alberta, **SK**-Saskatchewan, **MB**-Manitoba, **ON**-Ontario, **QC**-Quebec, **NB**-New Brunswick, **PE**-Prince Edward Island, **NS**-Nova Scotia, and **NF & LB**-Newfoundland and Labrador. The distribution records of all *Pityophthorus* species in Canada are presented according to Bright (1981), McNamara (1991), Wood and Bright (1992), and Bright and Skidmore (1997). The distribution of each *Pityophthorus* species was mapped by using the ARCGIS and ARCMAP software starting from an EXCEL database, which is available at the following web link: www.profs.inrs.ca/cguertin/ZOOKEYS_2013/MENU.html

## Results

To date, the following 17 *Pityophthorus* species have been recorded in the province of Quebec, Canada:

### Pityophthorus lautus

Eichhoff, 1872

http://species-id.net/wiki/Pityophthorus_lautus

Fig. 7

#### Records from Bright (1981).

**Aylmer**, **Communauté**-**Urbaine**-**de**-**l’Outaouais,** 14-VIII-1920, (3, CNC); **Wakefield**, **Les Collines**-**de**-**l’Outaouais**, 11-V-1951, (19, SLWC), *Rhus typhina*; **Sainte**-**Anne**-**de**-**Bellevue**, **Montréal**, ?, (10, CNC) / 2 individuals who supposedly originated from the same samples were found at LEMU and display an unreadable label.

#### New records.

**Montréal**, 14-V-1936, (9, ECLU); **Mont Saint**-**Hilaire**, **La**-**Vallée**-**du**-**Richelieu**, ?, (1, LFRC), **Mont Saint-Bruno**, **La**-**Vallée**-**du**-**Richelieu**, ?, (1, LFRC).

#### Distribution in Canada.

**NT**, **ON**, **QC**, **NB**, **NS**.

### Pityophthorus pulicarius

(Zimmermann, 1868)

http://species-id.net/wiki/Pityophthorus_pulicarius

Fig. 3

#### Records from Bright (1981).

**Chelsea**, **Les Collines**-**de**-**l’Outaouais**, 20-VI-1917, (6, CNC); **Grand-Remous**, **La**-**Vallée**-**de**-**la**-**Gatineau**, 17-VIII-1978, (2, LFRC), *Pinus banksiana*; **Wychwood**, **Communauté**-**Urbaine**-**de**-**l’Outaouais**,21-VI-1917, (2, CNC); **Pointe à David**, **La**-**Vallée**-**de**-**la**-**Gatineau**, 2-VI-1975, (1, LFRC), *Pinus banksiana*; **Lac Louvicourt**, **La**-**Vallée**-**de**-**l’Or**, 1-IX-1978, (2, LFRC), *Pinus banksiana*; **Moffet**, **Témiscamingue**, 16-VIII-1978, (2, LFRC), *Pinus banksiana*; **Rivière**-**aux**-**Rats**, **Le Haut**-**Saint**-**Maurice**, 14-VII-1978, (1, LFRC), *Pinus banksiana*; **Sainte**-**Anne**-**de**-**Bellevue**, **Montréal**, ?, (1, CNC).

#### New records.

**Chute**-**Saint**-**Philippe**, **Antoine**-**Labelle**, 22-VIII-1990, (1, MRNQ), *Pinus banksiana* / 11-IX-1990, (3, MRNQ), *Pinus banksiana*; **Chemin du Lac Petawaga**, **Antoine**-**Labelle**, 3-VII-1981, (1, MRNQ), *Pinus banksiana*; **L’Annonciation**, **Antoine**-**Labelle**, 30-VI-1985, (3, MRNQ), *Pinus resinosa*; **Lac Landron**, **La Vallée**-**de**-**la**-**Gatineau**, 5-VI-1982, (2, MRNQ), *Pinus banksiana*; **Baie Mercier**, **La Vallée**-**de**-**la**-**Gatineau**, 16-VIII-1979, (1, MRNQ), *Pinus banksiana*; **Lac Pageot, Pontiac**, 12-VII-1983, (1, MRNQ), *Pinus banksiana*; **Vinton**, **Pontiac**, 31-V-2011, (1, MRNQ), *Pinus resinosa*; **Chemin du Lac de l’Épine**, **La Vallée-de-l’Or**, 8-VII-1981, (1, MRNQ), *Pinus banksiana*; **Lac Quentin**, **La Vallée-de-l’Or**, 31-VII-1982, (5, MRNQ), *Pinus banksiana*; **Mont Saint-Michel**, **La-Vallée-de-l’Or**, 6-VII-2011, (1, MRNQ), *Pinus banksiana*; **Notre-Dame-du-Nord, Témiscamingue**, 16-VII-1985, (2, MRNQ), *Pinus resinosa*; **Guérin**, **Témiscamingue**, 31-VI-1982, (1, MRNQ), *Pinus banksiana*; **Lac Bend**, **Témiscamingue**, 21-VII-1981, (1, MRNQ), *Pinus sylvestris*; **Latulipe**, **Témiscamingue**, 24-VII-1981, (1, MRNQ), *Pinus banksiana*; **Lac Nodier**, **Témiscamingue**, 16-VII-1981, (1, MRNQ), *Pinus banksiana*; **Lac des Seize**, **Témiscamingue**, 13-VII-1983, (2, MRNQ), *Pinus banksiana*; **Saint**-**Bruno**-**de**-**Guigues**, **Témiscamingue**, 22-VII-2005, (1, MRNQ), *Pinus banksiana*; **Lac à Bédard**, **Témiscamingue**, 22-VII-1986, (1, MRNQ), *Pinus banksiana*; **Cloutier**, **Rouyn**-**Noranda**, 29-VII-1981, (2, MRNQ); *Pinus banksiana*; **Lac Bruyère**, **Rouyn**-**Noranda**, 20-VI-1983, (1, MRNQ), *Pinus banksiana*; **Lac Lavoie**, **Rouyn**-**Noranda**, 15-VII-1981, (1, MRNQ), *Pinus banksiana*; **Lac McWatters**, **Rouyn-Noranda**, 17-VII-1984, (2, MRNQ), *Pinus banksiana*; **La Morandière**, **Abitibi**, 13-VII-1984, (2, MRNQ), *Pinus banksiana*; **Villemontel**, **Abitibi**, 6-VII-1983, (4, LFRC), *Pinus banksiana*; **Lac Macamic**, **Abitibi Ouest**, 26-VI-1984, (1, MRNQ), *Pinus banksiana*; **Saint**-**Dominique**, **Abitibi Ouest**, 14-VII-2011, (1, MRNQ), *Pinus banksiana*; **Saint**-**Georges**, **Le Centre**-**de**-**la**-**Mauricie**, 22-VII-1981, (3, MRNQ), *Pinus resinosa*; **Saint**-**Antoine**-**Abbé**, **Le Haut**-**Saint**-**Laurent,** 22-V-2002, (2, MRNQ), *Pinus resinosa*; **Saint-Polycarpe**, **Vaudreuil-Soulange**, 5-VII-2002, (1, MRNQ), *Pinus strobus*; **Lac Wet**, **Le Haut**-**Saint**-**Maurice**, 13-VIII-1992, (1, MRNQ), *Pinus banksiana*; **Lefebvre**, **Drummond**, 28-V-1999, (4, MRNQ), *Pinus resinosa*; **Lac Roméo**, **Jamésie**, 20-VI-1981, (1, MRNQ), *Pinus banksiana*.

#### Distribution in Canada.

**SK**, **MB**, **ON**, **QC**, **NB**, **NS**.

### Pityophthorus nitidus

Swaine, 1917

http://species-id.net/wiki/Pityophthorus_nitidus

Fig. 4

#### Records from Bright (1981).

**Tullochgorum**, **Le Haut**-**Saint**-**Laurent**, 20-IX-1910, (2, CNC); **Sainte**-**Anne**-**de**-**Bellevue**, **Montreal**, ?, (2, CNC); **Saint**-**Gabriel**-**de**-**Rimouski**, **La Mitis,** 8-VII-1970, (6, CCC), *Picea glauca*.

#### New records.

**Les Étroits**, **Témiscouata**, 24-VII-1986, (2, MRNQ), *Pinus resinosa*; **Saint**-**Éleuthère**, **Témiscouata**, 2-VIII-1984, (11, MRNQ), *Picea glauca* / 1-VIII-1984, (2, MRNQ), *Pinus resinosa* / 17-VIII-1989, (3, MRNQ); **Estcourt**, **Témiscouata**, 1-VIII-1984, (2, MRNQ), *Pinus resinosa*; **Lac Nadreau**, **La Côte**-**de**-**Beaupré**, 9-VIII-1989, (16, MRNQ), *Picea mariana* / 31-VII-1990, (16, MRNQ), *Picea mariana*; **Saint-Alphonse-de-Caplan**, **Bonaventure**, 8-IX-1989, (1, MRNQ); **Saint-Elzéar**, **Bonaventure**, 27-VI-1984, (5, MRNQ), *Pinus sylvestris*; **Lac du Curé**, **Bonaventure**, 17-VI-1996, (4, MRNQ); **Rivington**, **Argenteuil**, 1-IX-1992, (10, MRNQ), *Pinus banksiana*; **Kinnear’s Mills**, **Les Appalaches**, 26-VIII-1993, (9, MRNQ), *Picea glauca* / 10-IX-1993, (3, MRNQ), *Picea glauca*; **Sacré**-**Cœur**-**de**-**Marie**, **Les Appalaches**, 5-VIII-1981, (5, MRNQ), *Picea abies*; **Routhierville**, **La Matapédia**, 31-VII-1996, (8,MRNQ), *Picea glauca*; **Saint**-**Paul**-**de**-**Montminy**, **Montmagny**, 24-VII-1981, (3, MRNQ), *Pinus resinosa*; **Saint**-**Joseph**-**de**-**Ham**, **Les Sources**, 21-VII-1981, (1, MRNQ), *Pinus resinosa*; **Les Éboulements**, **Charlevoix**, 4-VI-1981, (1, MRNQ), *Pinus strobus*; **Saint**-**Hilarion**, **Charlevoix**, 8-VIII-1990, (1, MRNQ), *Pinus strobus*; **Petite**-**Rivière**-**Saint**-**François**, **Charlevoix**, 19-VIII-1985, (2, MRNQ), *Picea abies*; **Lac Rivard**, **Maria**-**Chapdelaine**, 20-VII-2011, (1, MRNQ), *Picea mariana*; **Lac des Trois Élans**, **Maria**-**Chapdelaine**, 1-VIII-2001, (1, MRNQ), *Picea mariana*; **Pointe**-**Lebel**, **Manicouagan**, 7-VIII-1981, (1, MRNQ), *Pinus resinosa*; **Sainte**-**Eulalie**, **Nicolet-Yamaska**, 11-IX-1984, (2, MRNQ), *Picea abies*; **Saint**-**Thomas**-**de**-**Cherbourg**, **Matane**, 9-VIII-1979, (2, MRNQ), *Pinus banksiana*; **Ruisseau Ernest**, **Antoine**-**Labelle**, 6-VI-1981, (4, MRNQ), *Pinus strobus*; **Scotstown**, **Le Haut**-**Saint**-**François**, 26-VIII-1981, (3, MRNQ), *Picea glauca*; **Saint**-**Félix**-**d’Otis**, **Le Fjord**-**du**-**Saguenay**, 24-IX-1984, (6, MRNQ), *Pinus resinosa*; **Réservoir Baskatong**, **La Vallée-de-la-Gatineau**, 26-VI-1981, (2, MRNQ), *Pinus banksiana*; **Île d’Anticosti**, **Minganie**, 12-VI-1973, (4, CCC); **Longue**-**Pointe**-**de**-**Mingan**, **Minganie**, 25-VII-1980, (2, CCC); **Sept**-**Îles**, **Sept**-**Rivières**, 1-VIII-1982, (2, CCC), *Picea mariana* / 13-VI-1984, (1, CCC) / 8-VI-1985, (71, CCC) / 27-VII-1985, (1, CCC) / 3-VIII-1985, (30, CCC), *Picea mariana* / 24-V-1986, (5, CCC) / 6-VII-1986, (2, CCC) / 5-VIII-1986, (1, CCC) / 29-V-1987, (5, CCC) / 1-VI-1987, (1, CCC) / 7-VI-1987, (1, CCC) / 9-VII-1987, (2, CCC) / 16-VI-1988, (5, CCC) / 12-VII-1988, (1, CCC) / 2-VI-1990, (58, CCC) / 26-VII-1990, (1, CCC) / 23-VIII-1990, (16, CCC) / 10-VI-1991, (1, CCC) / 17-VII-1991, (11, CCC) / 20-VII-1991, (10, CCC) / 30-VII-1991, (1, CCC) / 6-VIII-1991, (4, CCC) / 9-VIII-1992, (4, CCC).

#### Distribution in Canada.

**NT**, **YT**, **AB**, **BC**, **ON**, **QC**, **NB**, **NS**, **NF** **&** **LB**.

### Pityophthorus intextus

Swaine, 1917

http://species-id.net/wiki/Pityophthorus_intextus

Fig. 9

#### New records.

**Saint-Hilarion**, **Charlevoix**, 8-VIII-1990, (1, MRNQ), *Picea glauca*; **Lac Fourcet**, **Antoine-Labelle**, 9-VII-1981, (1, MRNQ), *Picea mariana*; **Saint-Hérménégilde**, **Coaticook**, 5-VII-1985, (1, MRNQ), *Pinus resionsa*; **Lac Poutrincourt**, **Le Domaine-du-Roy**, 25-VII-2007, (1, MRNQ), *Pinus banksiana*; **Sept-Îles**, **Sept-Rivières**, 13-VII-1987, (1, CCC) / 16-VI-1988, (1, CCC).

#### Distribution in Canada.

**BC**, **AB**, **SK**, **MB**, **ON**, **QC**, **NB**, **NS**, **NF** **&** **LB**.

### Pityophthorus pulchellus pulchellus

Eichhoff, 1869

http://species-id.net/wiki/Pityophthorus_pulchellus_pulchellus

Fig. 2

#### Records from Bright (1981).

**Kazabazua**, **La**-**Vallée**-**de**-**la**-**Gatineau**, 13-XII-1917, (5, CNC), *Pinus banksiana*; **Lac Saint**-**Jean, Lac Saint**-**Jean E**, ?, (6, LFRC), *Pinus banksiana*; **Sainte-Anne-du-Lac (Zec Mitchinamécus)**, **Antoine**-**Labelle**, 4-VII-1978, (48, LFRC), *Pinus banksiana*.

#### Records from Paquin and Dupérré (2001).

**Jamésie**, 14-IX-1997, (1, LEMU), *Picea mariana*.

#### New records.

**Sainte**-**Françoise**, **Bécancour**, 18-VI-1986, (4, MRNQ), *Pinus strobus*; **Colombier** (**Serres**), **La Haute**-**Côte**-**Nord**, 16-VII-1986, (2, MRNQ), *Pinus resinosa*; **Les Escoumins**, **La Haute**-**Côte**-**Nord**, 6-VI-1984, (1, CCC); **Saint**-**Ambroise**, **Le Fjord**-**du**-**Saguenay**, 6-VII-1992, (1, MRNQ), *Pinus strobus*; **Lac du Grand** **Détour**, **Le Fjord**-**du**-**Saguenay**, 20-VII-2011, (1, MRNQ), *Pinus banksiana*; **Saint**-**David**-**de**-**Falardeau**, **Le Fjord**-**du**-**Saguenay**, 9-VII-1996, (1, MRNQ), *Pinus resinosa*; **Sept**-**Îles**, **Sept**-**Rivières**, 8-VI-1985, (4, CCC) / 16-V-1986, (3, CCC) / 6-VII-1986 (1, CCC) / 29-V-1987, (1, CCC) / 17-VI-1987, (1, CCC) / 25-VI-1987, (2, CCC) / 13-VII-1987, (15, CCC) / 12-VI-1988, (3, CCC) / 21-VI-1988, (7, CCC) / 4-VII-1988, (3, CCC) / 19-V-1989, (1, CCC) / 9-VI-1989, (1, CCC) / 27-V-1990, (7, CCC) / 2-VI-1990, (99, CCC) / 26-VII-1990, (1, CCC) / 27-VIII-1990, (3, CCC) / 29-V-1991, (22, CCC) / 12-VIII-1991, (1, CCC) / 22-V-1992, (2, CCC); **Lac Saint**-**Ludger**, **Lac Saint**-**Jean**-**Est**, 18-VII-1981, (3, MRNQ), *Pinus banksiana*;**Ruisseau du Pont**, **Lac Saint**-**Jean**-**Est**, 31-VII-1984, (10, MRNQ), *Pinus banksiana*; **Notre**-**Dame**-**du**-**Rosaire**, **Lac Saint**-**Jean**-**Est**, 1-VII-1981, (1, MRNQ), *Picea glauca*; **Lac Fourcet**, **Antoine**-**Labelle**, 9-VII-1981, (1, MRNQ), *Picea mariana*; **Chute**-**Saint**-**Philippe**, **Antoine**-**Labelle**, 16-VI-1995, (4, MRNQ), *Pinus banksiana*; **Landrienne**, **Abitibi**, 15-VII-1987, (2, MRNQ), *Pinus banksiana*; **Authier Nord**, **Abitibi Ouest**, 13-VII-1979, (1, MRNQ), *Pinus banksiana*; **La Morandière, Abitibi**, 11-VII-1984, (9, MRNQ), *Pinus banksiana*; **Guyenne Township, Abitibi**, 16-VIII-1983, (1, MRNQ), *Pinus banksiana*; **Lac Castagnier**, **Abitibi**, 9-IX-1986, (3, MRNQ), *Pinus banksiana*; **Villemontel**, **Abitibi**, 31-VIII-1983, (80, LFRC), *Pinus banksiana*; **Lac Dubois**, **Témiscamingue**, 21-VIII-1986, (1, MRNQ), *Pinus banksiana*; **Lorainville**, **Témiscamigue**, 4-VI-1981, (51, MRNQ), *Pinus banksiana*; **Lac Nodier**, **Témiscamingue**, 4-VIII-1981, (1, MRNQ), *Pinus banksiana*; **Cloutier,** **Rouyn**-**Noranda**, 30-VII-1981, (4, MRNQ), *Pinus banksiana*; **Lac Surimau**, **Rouyn-Noranda**, 11-VIII-1982, (5, MRNQ), *Pinus banksiana*; **Rapide**-**Deux**, **Rouyn**-**Noranda**, 6-VI-1983, (1, MRNQ), *Pinus banksiana*;**Lac Charles,** **Le Haut**-**Saint**-**Maurice**, 17-VII-1987, (1, MRNQ), *Pinus banksiana*; **Lac Wet**, **Le Haut**-**Saint**-**Maurice**, 13-VIII-1992, (15, MRNQ), *Pinus banksiana*; **Lac Gosselin**, **Le Haut**-**Saint**-**Maurice**, 1-VIII-1981, (6, MRNQ), *Pinus banksiana*; **Lac Louvicourt**, **La**-**Vallée**-**de**-**l’Or**, 6-IX-1984, (1, MRNQ), *Pinus banksiana*; **Lac Tremblay**, **La**-**Vallée**-**de**-**l’Or,** 27-VII-2011, (1, MRNQ), *Pinus banksiana*; **Rivière Mégiscane**, **La**-**Vallée**-**de**-**l’Or**, 12-VI-1981, (5, MRNQ), *Pinus banksiana*; **Lac Faillon**, **La**-**Vallée**-**de**-**l’Or,** 16-VI-1981, (2, MRNQ), *Pinus banksiana*; **Lac Villebon, La**-**Vallée**-**de**-**l’Or**, 6-VI-1981, (4, MRNQ), *Pinus banksiana* / 2-IX-1981, (4, MRNQ), *Pinus banksiana*; **Lac Fournière**, **La**-**Vallée**-**de**-**l’Or**, 4-VIII-1981, (3, MRNQ), *Pinus banksiana* / 7-VIII-1981, (3, MRNQ), *Pinus banksiana*; **Lac Prospère**, **La**-**Vallée**-**de**-**l’Or**, 22-VIII-1981, (3, MRNQ), *Pinus banksiana*; **Lac Palouse**, **La**-**Vallée**-**de**-**l’Or**, 9-VI-1983, (26, MRNQ), *Pinus banksiana*; **Pointe**-**Lebel Airport**, **Manicouagan**, 17-VIII-1981, (2, MRNQ), *Pinus resinosa*; **Lac Pistuacanis**, **Manicouagan**, 18-VII-1981, (4, MRNQ), *Pinus banksiana*; **Rivière Betsiamites**, **Manicouagan**, 5-VIII-1981, (1, MRNQ), *Pinus banksiana*; **Lac Saint**-**Pierre**, **Le Domaine**-**du**-**Roy**, 17-VI-1981, (4, MRNQ), *Pinus banksiana*; **Saint**-**Félicien**, **Le Domaine**-**du**-**Roy**, 26-V-2006, (5, MRNQ), *Pinus banksiana*; **Lac Beemer**, **Le Domaine**-**du**-**Roy**, 18-VII-2007, (2, MRNQ), *Pinus banksiana*; **Lac Mignault**, **Le Domaine**-**du**-**Roy**, 15-IX-2005, (1, MRNQ), *Picea* sp.; **Rivière Désert**, **La Vallée**-**de**-**la**-**Gatineau**, 12-VII-1984, (1, MRNQ), *Pinus strobus*; **Lac Rond**, **La Vallée**-**de**-**la**-**Gatineau**, 22-VI-1983, (52, MRNQ), *Pinus banksiana*; **Lac des Outaouais**, **La Vallée**-**de**-**la**-**Gatineau**, 18-VIII-1981, (6, MRNQ), *Pinus banksiana*; **Lac Mosher**, **La Vallée**-**de**-**la**-**Gatineau**, 26-VII-1983, (9, MRNQ), *Pinus banksiana*; **Lac Danford**, **La Vallée**-**de**-**la**-**Gatineau**, 16-VIII-1980, (3, MRNQ), *Pinus banksiana* / 13-IX-1983, (3, MRNQ), *Pinus resinosa*; **Kazabazua**, **La Vallée**-**de**-**la**-**Gatineau**, 13-XII-1971, (4, MRNQ), *Pinus resinosa* / 28-V-1984 (7, MRNQ), *Pinus resinosa*; **Lac Dickson**, **Pontiac**, 16-VII-1992, (1, MRNQ), *Pinus banksiana*; **Lac Nigault**, **Pontiac**, 29-VIII-1994, (2, MRNQ), *Pinus resinosa*; **Lac Charrette**, **Pontiac**, 5-VIII-1981, (2, MRNQ), *Pinus strobus*; **Bas**-**de**-**l’Anse**, **Charlevoix**-**Est**, 18-VI-1981, (2, MRNQ), *Pinus sylvestris*; **Lac Port au Saumon**, **Charlevoix**-**Est**, 23-VII-1981, (2, MRNQ), *Pinus banksiana*; **Saint**-**Sébastien**-**de**-**Frontenac**, **Le Granit**, 16-VII-1981, (4, MRNQ), *Pinus banksiana*; **Lac Rivaille**, **Maria**-**Chapdelaine**, 28-VIII-2008, (6, MRNQ), *Pinus banksiana*; **Bromptonville**, **Sherbrooke**, 31-VII-1981, (1, MRNQ), *Pinus sylvestris*; **Lac Winsch**, **Jamésie**, 12-VIII-1981, (1, MRNQ), *Pinus banksiana*; **Les Étroits**, **Témiscouata**, 24-VII-1986, (3, MRNQ), *Pinus resinosa*.

#### Distribution in Canada.

**YT**, **NT**, **BC**, **AB**, **SK**, **MB**, **ON**, **QC**, **NB**.

### Pityophthorus cariniceps

LeConte & Horn, 1876

http://species-id.net/wiki/Pityophthorus_cariniceps

Fig. 5

#### Records from Bright (1981).

**Sainte**-**Anne**-**de**-**Bellevue**, **Montréal,** 17-VIII-1910, (1, CNC), *Pinus* sp.; **L**’**Île Perrot**, **Vaudreuil**-**Soulanges**, various dates, (42, CNC) / 4 specimens supposedly originating from the same samples were found at LEMU, *Pinus* sp.; **Old Chelsea**, **Les Collines**-**de**-**l’Outaouais**, 23-VI-1966, (14, CNC), *Pinus strobus*; **Wychwood**, **Communauté**-**Urbaine**-**de**-**l’Outaouais**, 2-VI-1917, (5, CNC), *Pinus resinosa*.

#### New records.

**Ways**-**Mills**, **Coaticoock**, 13-V-1988, (1, MRNQ), *Pinus strobus*; **Beebe Plain**, **Memphrémagog**, 5-VI-1989, (1, MRNQ), *Pinus strobus*; **Saint**-**Aimé**-**des**-**Lacs**, **Charlevoix**-**Est**, 26-VI-1992, (4, MRNQ), *Pinus strobus*; **Shawville**, **Pontiac**, 9-V-2001, (3, MRNQ), *Pinus resinosa*; **Lac Hickey**, **Pontiac**, 14-V-2007, (2, MRNQ), *Pinus strobus*; **Lac Prendergast**, **Pontiac**, 25-V-1981, (1, MRNQ), *Pinus strobus*; **Thorne Centre**, **Pontiac**, 26-V-2003, (2, MRNQ), *Pinus resinosa*; **Baie du Chat**, **Pontiac**, 5-VII-1981, (5, MRNQ), *Pinus strobus*; **Fort Coulonge**, **Pontiac**, various dates in the period 2008-2012, (124, INRS-IAF), *Pinus strobus*; **Lac Cayamant, Pontiac**, various dates in the period 2008-2012, (22, INRS-IAF), *Pinus strobus*; **Lac Ruthledge**, **Les Collines**-**de**-**l’Outaouais**, 18-VII-1981, (2, MRNQ), *Pinus strobus*; **Sainte**-**Cécile**-**de**-**Masham**, **Les Collines**-**de**-**l’Outaouais**, 31-VII-1972, (10, LFRC), *Pinus sylvestris*; **Saint**-**Charles**-**de**-**Mandeville**, **D’Autray**, 13-VII-1981, (5, MRNQ), *Pinus strobus*; **Saint**-**Zéphirin**, **Nicolet**-**Yamaska**, 29-VI-1981, (4, MRNQ), *Pinus strobus*; **Les Éboulements**, **Charlevoix**, 4-VI-1981, (2, MRNQ), *Pinus strobus*; **Camp l’Oasis**, **Portneuf**, 12-VI-1980, (1, MRNQ), *Pinus strobus*; **Island Brook**, **Les Haut**-**Saint**-**François**, 30-VII-1981, (1, MRNQ), *Pinus resinosa*; **Cookshire**, **Le Haut**-**Saint**-**François**, 26-IV-2002, (2, MRNQ), *Pinus banksiana*; **Lemieux**, **Bécancour**, 27-VII-1981, (2, MRNQ), *Pinus banksiana*; **Zec** **Chauvin**, **La Haute**-**Côte**-**Nord**, 13-IV-1983, (5, MRNQ), *Pinus strobus* / 8-VI-1983, (2, MRNQ), *Pinus strobus*; **Mont**-**Saint**-**Hilaire**, **La**-**Vallée**-**du**-**Richelieu**, 24-V-1916, (1, LEMU), *Pinus strobus*; **Oka**, **Deux**-**Montagnes**, 5-VII-1978, (1, CCC), *Pinus strobus*; **Saint**-**Amable**, **Marguerite**-**D’Youville**, 11-V-1999, (3, MRNQ), *Pinus strobus* / various dates in the period 2008-2012, (1051, INRS-IAF), *Pinus strobus*; **Saint**-**Claude**, **Le**-**Val**-**Saint**-**François**, various dates in the period 2008-2012, (85, INRS-IAF), *Pinus strobus*; **Saint**-**Simon**-**les**-**Mines**, **Beauce**-**Sartigan**, various dates in the period 2008-2012, (93, INRS-IAF), *Pinus strobus*; **Cap-Tourmente**, **La Côte**-**de**-**Beaupré**, various dates in the period 2008-2012, (178, INRS-IAF), *Pinus strobus*; **Sept**-**Îles**, **Sept**-**Rivières**, 8-VI-1985, (1, CCC) / 12-VII-1986, (1, CCC) / 13-VI-1987, (1, CCC) / 10-VII-1987, (1, CCC) / 18-VI-1988, (1, CCC) / 21-VI-1988, (1, CCC) / 4-VII-1988, (2, CCC) / 27-V-1990, (1, CCC) / 29-V-1991, (3, CCC) / 29-VII-1991, (1, CCC); **Lac Ramsay**, **Les Collines**-**de**-**l’Outaouais**, 18-V-2009, (1, CCC); **Johnville**, **Compton**, 13-V-1987 (1, CLC) / 17-V-1987 (2, CLC) / 20-V-1987 (1, CLC) / 22-V-1988 (1, CLC) / 17-V-1989 (3, CLC) / 21-V-1989 (1, CLC) / 22-X-1989 (1, CLC).

#### Distribution in Canada.

**AB**, **SK**, **MB**, **ON**, **QC**, **NB**, **NS**.

### Pityophthorus biovalis

Blackman, 1922

http://species-id.net/wiki/Pityophthorus_biovalis

Fig. 6

#### New records.

**L’Annonciation**, **Antoine**-**Labelle**, 20-VII-1992, (4, MRNQ), *Pinus resinosa*; **Saint**-**Philémon**, **Bellechasse**, 29-V-1981, (4, MRNQ), *Pinus banksiana* / 20-VII-1981, (5, MRNQ), *Picea abies*; **Lac du Port au Saumon**, **Charlevoix**-**Est**, 23-VII-1981, (3, MRNQ), *Pinus banksiana*; **Saint**-**Claude**, **Le Val**-**Saint**-**François**, 20-VII-1992, (5, MRNQ), *Picea glauca* / 28-VII-2008, (2, INRS-IAF), *Pinus strobus* / 9-VI-2011, (5, INRS-IAF), *Pinus strobus*; **Quatre**-**Chemins**, **Les Pays**-**d’en**-**Haut**, 18-V-1993, (4, MRNQ), *Pinus strobus*; **Woburn**, **Le Granit**, 9-VII-1981, (2, MRNQ), *Pinus resinosa*; **Lac Cayamant**, **La**-**Vallée**-**de**-**la**-**Gatineau**,15-VII-2009, (1, INRS-IAF), **Dosquet**, **Lotbinière**, 20-V-1972, (1, CCC); **Saguenay**, **Le Fjord**-**du**-**Saguenay**, 6-VIII-1984, (1, CCC), *Pinus banksiana*.

#### Distribution in Canada.

**ON**, **QC**, **NS**.

### Pityophthorus carinatus carinatus

Bright, 1978

http://species-id.net/wiki/Pityophthorus_carinatus_carinatus

Fig. 9

#### Record from Bright (1981):

**Sainte**-**Anne**-**du**-**Lac**, **Antoine**-**Labelle**, 4-VII-1978, (2, CNC), *Pinus strobus*.

#### New records.

**Lac Needham**, **Pontiac**, 12-VII-2001, (4, MRNQ), *Pinus strobus*; **Saint**-**Augustin**, **Maria**-**Chapdelaine**, 28-VIII-1981, (1, MRNQ), *Pinus banksiana*; **Notre**-**Dame**-**du**-**Rosaire**, **Lac Saint**-**Jean**-**Est**, 26-V-1981, (3, MRNQ), *Picea glauca*; **Boilleau**, **Papineau**, 29-VII-2004, (1, MRNQ), *Picea mariana*; **Cookshire**, **Compton**, 23-V-2000, (2, LFRC), *Pinus resinosa*; **Johnville**, **Compton**, 24-V-1987, (1, CLC) / 17-V-1987, (1, CLC) / 18-V-1988, (1, CLC) / 21-V-1989 (1, CLC) / 11-VI-1989, (1, CLC); **Dosquet**, **Lotbinière**, 16-V-1976, (5, CCC); **Lac Cayamant**, **La**-**Vallée**-**de**-**la**-**Gatineau**,17-VI-2008, (1, INRS-IAF); **Fort-Coulonge**, **Pontiac**, 16-VI-2011, (1, INRS-IAF).

#### Distribution in Canada.

**QC**, **NB**.

### Pityophthorus balsameus

Blackman, 1922

http://species-id.net/wiki/Pityophthorus_balsameus

Fig. 7

#### New records.

**Cap**-**Saint**-**Ignace**, **Montmagny**, 26-VIII-1998, (2, MRNQ), *Picea abies* / 4-IX-1998, (2, MRNQ), *Picea abies*; **Ways**-**Mills**, **Coaticook**, 13-V-1988, (5, MRNQ), *Pinus strobus*; **Doncaster Township**, **Les Laurentides**, 7-VII-1981, (1, MRNQ), *Pinus resinosa*; **Saint**-**Philémon**, **Bellechasse**, 29-V-1981, (1, MRNQ), *Pinus resinosa*; **Armagh**, **Bellechasse**, 16-IX-1979, (1, MRNQ), *Pinus resinosa*; **Sainte**-**Marguerite**, **La Nouvelle**-**Beauce**, 6-VII-1981, (4, MRNQ), *Picea abies*; **Mitchell Township**, **La**-**Vallée**-**de**-**la**-**Gatineau**, 26-VI-1981, (1, MRNQ), *Pinus banksiana*; **Rivière Petit Saguenay**, **Le Fjord**-**du**-**Saguenay**, 25-V-1982, (5, MRNQ), *Pinus resinosa*; **Saint**-**Ferréol**-**des**-**Neiges**, **La**-**Côte**-**de**-**Beaupré**, 3-VII-1990, (1, MRNQ), *Pinus resinosa*; **Saint**-**Joachim**-**de**-**Courval**, **Drummond**, 26-VII-2001, (2, MRNQ), *Picea abies*; **Saint**-**Germain**-**de**-**Grantham**, **Drummond**, 24-VII-1981, (1, MRNQ), *Pinus banksiana*; **Sacré**-**Cœur**, **La Haute**-**Côte**-**Nord**, 8-VII-1992, (2, MRNQ), *Pinus banksiana*; **Notre**-**Dame**-**du**-**Rosaire**, **Lac Saint**-**Jean**-**Est**, 26-V-1981, (1, MRNQ), *Picea glauca*; **Valcartier**, **La Jacques**-**Cartier**, 3-VIII-1981, (1, MRNQ), *Pinus resinosa*; **Dosquet**, **Lotbinière**, 9-VI-1970, (1, CCC) / 28-IV-1984, (1, CCC).

#### Distribution in Canada.

**NT**, **ON**, **QC**, **NB**, **NS**.

### Pityophthorus briscoei

Blackman, 1922

http://species-id.net/wiki/Pityophthorus_briscoei

Fig. 6

#### Record from Bright (1981).

**Sainte**-**Anne**-**du**-**Lac**, **Antoine**-**Labelle**, 4-VII-1978, (1, LFRC), *Pinus strobus*.

#### New records:

**Villette**, **Coaticook**, 18-VII-1985, (6, MRNQ), *Pinus sylvestris*; **Saint**-**Hilarion**, **Charlevoix**, 8-VIII-1990, (1, MRNQ), *Picea glauca*; **Saint**-**Herménégilde**, **Coaticook**, 8-VII-1985, (3, MRNQ), *Pinus resinosa*; **Poupore**, **Les Collines**-**de**-**l’Outaouais**, 30-VI-1992, (4, MRNQ), *Pinus banksiana*; **Simpson Township**, **Drummond**, 19-VIII-1981, (4, MRNQ), *Picea glauca*; **Saint**-**Joachim**-**de**-**Courval**, **Drummond**, 20-VII- 2001, (2, MRNQ), *Picea glauca*; **Island Brook**, **Le Haut**-**Saint**-**François**, 30-VII-1981, (4, MRNQ), *Pinus resinosa*; **Rawdon Township**, **Matawinie**, 28-VII-1973, (17, MRNQ), *Pinus resinosa*; **Valcartier**, **La Jacques**-**Cartier**, 23-VII-1991, (3, LFRC), *Pinus strobus*.

#### Distribution in Canada:

**ON**, **QC**, **NB**.

### Pityophthorus concavus

Blackman, 1928

http://species-id.net/wiki/Pityophthorus_concavus

Fig. 8

#### Record from Bright (1981).

**Kazabazua**, **La** **Vallée**-**de**-**la**-**Gatineau**, 24-VII-1966, (2, CNC), *Pinus banksiana*.

#### New records.

**Cap**-**Saint**-**Ignace**, **Montmagny**, 4-IX-1998, (2, MRNQ), *Picea abies* / 9.VII.2002, (1, MRNQ), *Picea rubens*; **Armagh**, **Bellechasse**, 16-IX-1979, (1, MRNQ), *Pinus resinosa*; **Doncaster Township**, **Les Laurentides**, 7-VII-1981, (1, MRNQ), *Pinus resinosa*.

#### Distribution in Canada.

**ON**, **QC**, **NB**, **NS**.

### Pityophthorus ramiperda

Swaine, 1917

http://species-id.net/wiki/Pityophthorus_ramiperda

Fig. 9

#### Records from Bright (1981).

**L**’**Île Perrot**, **Vaudreuil**-**Soulanges**, 30-VII-1910, (1, CNC), *Pinus strobus*; **Sainte**-**Anne**-**de**-**Bellevue**, **Montréal**, 11-VIII-1911, (1, CNC), *Pinus strobus*.

#### Record from Paquin and Dupérré (2001).

**Jamésie**, 15-VI-1997, (1, LEMU), *Picea mariana*.

#### New records.

**Paul**-**Sauvé Park, Deux Montagnes**, 27-VII-1982, (4, LFRC), *Pinus strobus*; **Île**-**du**-**Grand**-**Calumet**, **Pontiac**, 31-VIII-1983, (3, LFRC), *Pinus strobus*.

#### Distribution in Canada.

**ON**, **QC, NS**.

### Pityophthorus opaculus

LeConte, 1878

http://species-id.net/wiki/Pityophthorus_opaculus

Fig. 8

#### Records from Bright (1981).

**Sainte**-**Anne**-**de**-**Bellevue**, **Montréal**, ?, (2, CNC); **Gaspé Co**., 2-VIII-1933, (5, CNC), *Picea glauca*; **Hudson**, **Vaudreuil**-**Soulanges**, 6-V-1910, (1, CNC), *Larix* sp.

#### Records from Paquin and Dupérré (2001).

**Jamésie**, 15-VI-1997, (7, LEMU) and (2, ROUM) *Picea mariana* / 22-VI-1997, (2, LEMU) and (2, ROUM), *Picea mariana* / 29-VI-1997, (2, LEMU) and (5, ROUM), *Picea mariana* / 6-VII-1997, (2, LEMU) and (1, ROUM), *Picea mariana* / 20-VII-1997, (2, ROUM), *Picea mariana* / 3-VIII-1997, (1, LEMU), *Picea mariana* / 10-VIII-1997, (1, ROUM), *Picea mariana* / 24-VIII-1997, (3, LEMU), *Picea mariana* / 7-IX-1997, (1, ROUM), *Picea mariana* / 28-IX-1997, (1, LEMU) and (1, ROUM), *Picea mariana*; **Lac Duparquet**, **Abitibi**, 12-VI-1994, (1, ROUM), *Cedrus* sp. / 22-VII-1997, (1, ROUM); **Lac Labyrinthe**, **Témiscamingue**, 21-VII-1996, (1, ROUM), *Abies* sp. / 18-VIII-1996, (1, ROUM), *Cedrus* sp. and *Abies* sp. stand.

#### New records.

**Lac Hubbard**, **La Vallée**-**de**-**la**-**Gatineau**, 31-VII-2001, (3, MRNQ), *Picea mariana*; **Lac Ollivon, La Vallée**-**de**-**l’Or**, 12-VIII-2002, (3, MRNQ), *Picea mariana*; **Gentilly**, **Bécancour**, 8-VII-2002, (1, MRNQ), *Picea glauca*; **Petite-Rivière-Saint-François**, **Charlevoix**, 19-VIII-1985, (1, MRNQ), *Picea abies*; **Valcartier**, **La Jacques**-**Cartier**, 13-VIII-1981, (1, MRNQ), *Pinus resinosa*; **Fort-Coulonge**, **Pontiac**, 25-V-2009, (1, INRS-IAF) / 16-VI-2011, (2, INRS-IAF), *Pinus strobus* / 16-VIII-2011, (2, INRS-IAF), *Pinus strobus*; **Saint**-**Claude**, **Le**-**Val**-**Saint**-**François**, 7-VII-2011, (1, INRS-IAF), *Pinus strobus*; **Sept**-**Îles**, **Sept**-**Rivières**, 29-V-1987, (1, CCC) / 15-VIII-1987, (1, CCC) / 12-VI-1988, (1, CCC) / 21-VI-1988, (1, CCC) / 2-VI-1990, (1, CCC) / 26-VII-1990, (1, CCC) / 23-VIII-1990, (1, CCC) / 6-VIII-1991, (11, CCC) / 9-VIII-1991, (1, CCC) / 13-VIII-1991, (1, CCC).

#### Distribution in Canada.

**YK**, **NT**, **AB**, **BC**, **SK**, **MB**, **ON**, **QC**, **NB**, **NS**, **NF** **&** **LB**.

### Pityophthorus dentifrons

Blackman, 1922

http://species-id.net/wiki/Pityophthorus_dentifrons

Fig. 7

#### Records from Bright (1981).

**Gaspé** Co., 2-VIII-1933, (10, CNC), *Picea glauca*.

#### New records.

**Sainte-Eulalie**, **Nicolet**-**Yamaska**, 11-IX-1984, (2, MRNQ), *Picea* sp., **Fort- Coulonge**, **Pontiac**, 25-V-2009, (1, INRS-IAF), *Pinus strobus*; **Saint**-**Simon**-**les**-**Mines**, **Beauce**-**Sartigan**, 30-VI-2009, (1, INRS-IAF), *Pinus strobus*.

#### Distribution in Canada.

**AB**, **ON**, **QC**, **NB**, **PE**, **NS**, **NF** **&** **LB**.

### Pityophthorus puberulus

(LeConte, 1868)

http://species-id.net/wiki/Pityophthorus_puberulus

Fig. 1

#### Records from Bright (1981).

**Kazabazua**, **La** **Vallée**-**de**-**la**-**Gatineau**, 24-VIII-1966, (3, CNC), *Pinus banksiana*; **Campbell’s Bay**, **Pontiac**, 24-VI-1978, (1, CCC); **Sainte**-**Anne**-**de**-**Bellevue, Montréal**,? , 1910, (15, CNC), *Pinus* sp. / one specimen supposedly originating from the same samples was found at LEMU; **Sainte**-**Marie**-**de**-**Beauce**, **La** **Nouvelle**-**Beauce**, ?-VIII-1975, (6, LFRC), *Pinus banksiana*.

#### New records.

**Saint**-**Théodore**, **Matawinie**, 25-VII-1984, (6, MRNQ), *Pinus resinosa*; **Réservoir** **Taureau**, **Matawinie**, 10-VII-1981, (2, MRNQ), *Pinus resinosa*; **Baldwin**-**Mills**, **Coaticook**, 8-VII-1985, (2, MRNQ), *Pinus sylvestris*; **Sainte**-**Edwidge**, **Coaticook**, 8-VIII-1990, (2, MRNQ), *Pinus sylvestris*; **Saint-Herménégilde**, **Coaticook**, 8-VII-1985, (6, MRNQ), *Pinus sylvestris*; **Trois**-**Lacs**, **Le Granit**, 24-VII-1986, (10, MRNQ), *Pinus resinosa* / 29-VII-1987, (3, MRNQ), *Pinus resinosa*; **Notre**-**Dame**-**des**-**Bois**, **Le Granit**, 8-VIII-1978, (7, MRNQ), *Pinus strobus* / 26-VII-1979, (5, MRNQ), *Pinus resinosa*; **Clinton Township**, **Le Granit**, 4-VI-1981, (3, MRNQ), *Pinus sylvestris*; **Chénéville**, **Papineau**, 5-VIII-1987, (4, MRNQ), *Pinus resinosa*; **Ripon**, **Papineau**, 8-VII-1992, (1, MRNQ), *Pinus banksiana*; **Notre**-**Dame**-**de**-**la**-**Paix**, **Papineau**, 16-VII-1993, (2, MRNQ), *Pinus resinosa*; **Lac** **Quatre Chemins**, **Papineau**, 1-IX-1983, (3, MRNQ), *Pinus resinosa*; **Charteris**, **Pontiac**, 24-V-2000, (2, MRNQ), *Pinus sylvestris* / 6-VI-2000, (2, MRNQ), *Pinus banksiana*; **Thorne**, **Pontiac**, 24-V-2000, (1, MRNQ), *Pinus resinosa*; **Lac Prendergast**, **Pontiac**, 19-VI-1980, (1, MRNQ), *Pinus strobus*; **Lac de la Ferme**, **Pontiac**, 12-VI-1980, (1, MRNQ), *Pinus resinosa*; **Vinton, Pontiac**, 31-V-2011, (2, MRNQ), *Pinus banksiana*; **Lac Lacaille**, **Les Collines**-**de**-**l’Outaouais**, 30-VIII-1981, (2, MRNQ), *Pinus resinosa*; **Lac Hamilton**, **Les Collines**-**de**-**l’Outaouais**, 26-VIII-1983, (2, MRNQ), *Pinus resinosa*; **Lac de la Grande Fourche**, **Rivière**-**du**-**Loup**, 6-VIII-1979, (4, MRNQ), *Pinus resinosa*; **Saint**-**Pierre**-**de**-**Lamy**, **Témiscouata**, 25-VII-1985, (4, MRNQ), *Pinus resinosa*; **Cookshire**, **Le Haut**-**Saint**-**François**, 11-VIII-1999, (1, MRNQ), *Pinus strobus* / 23-V-2000, (2, LFRC), *Pinus resinosa* / 30-V-2000, (1, LFRC), *Pinus resinosa* / 14-VI-2011, (1, LFRC), *Pinus resinosa*; **Scotstown**, **Le Haut**-**Saint**-**François**, 23-IX-1999, (1, MRNQ), *Pinus sylvestris*; **Bishopton**, **Le Haut**-**Saint**-**François**, 23-V-2001, (1, LFRC), *Pinus* sylvestris / 30-V-2011, (1, LFRC), *Pinus* resinosa / 24-V-2011, (1, LFRC), *Pinus resinosa*; **Waterville**, **Compton**, 17-V-2011, (1, LFRC), *Pinus resinosa* / 24-V-2011 (3, LFRC), *Pinus resinosa* / 7-VI-2011, (1, LFRC), *Pinus resinosa* / 14-VI-2011, (4, LFRC), *Pinus resinosa*; **Johnville**, **Compton**, 14-VI-2011, (1, LFRC), *Pinus resinosa* / 27-V-1987 (1, CLC) / 21-VI-1987 (1, CLC) / 19-VII-1987 (1, CLC) / 11-V-1988 (1, CLC) / 22-V-1988 (1, CLC) / 29-V-1988 (1, CLC) / 1-VI-1988 (1, CLC) / 6-VII-1988 (1, CLC) / 21-V-1989 (1, CLC) / 24-V-1989 (1, CLC) / 25-VI-1989 (2, CLC) / 6-VII-1989 (1, CLC) / 9-VII-1989 (1, CLC) / 16-VII-1989 (1, CLC) / 21-IX-1989 (1, CLC); **Huntingville**, **Sherbrooke**, 14-VI-2011, (4, LFRC), *Pinus resinosa*; **Sainte**-**Marie**-**de**-**Blandford**, **Bécancour**, 29-VI-1981, (2, MRNQ), *Pinus resinosa*; **Lemieux**, **Bécancour**, 27-VII-1981, (3, MRNQ), *Pinus banksiana*; **Sainte**-**Séraphine**, **Arthabaska**, 5-VIII-1993, (2, MRNQ), *Pinus resinosa*; **Victoriaville**, **Arthabaska**, 4-X-1978, (10, MRNQ), *Pinus resinosa*; **Saint**-**Didace**, **D’Autray**, 19-VIII-1978, (1, MRNQ), *Pinus resinosa*; **Berthierville**, **D’Autray**, 4-IX-1997, (2, MRNQ), *Pinus resinosa* / 27-V-1976, (5, CCC), *Pinus strobus*; **Ruisseau Sainte**-**Émilie**, **D’Autray**, 19-VIII-1979, (1, MRNQ), *Pinus resinosa*; **Bromptonville**, **Le Val**-**Saint**-**François**, 31-VII-1981, (3, MRNQ), *Pinus resinosa*; **Saint**-**Adelphe**, **Mékinac**, 30-VII-1986, (5, MRNQ), *Pinus resinosa*; **Lac Éclairé**, **Mékinac**, 30-VII-1980, (2, MRNQ), *Pinus resinosa*; **Saint**-**Pierre**-**Montmagny**, **Montmagny**, 7-VIII-1979, (3, MRNQ), *Pinus banksiana*; **Lac Morigeau**, **Montmagny**, 31-VII-2000, (3, MRNQ), *Pinus resinosa*; **Saint**-**David**-**de**-**Falardeau**, **Le Fjord**-**du**-**Saguenay**, 8-VII-2000, (1, MRNQ), *Pinus resinosa*; **Ferland**-**et**-**Boilleau**, **Fjord**-**du**-**Saguenay**, 18-VII-1988, (1, MRNQ), *Pinus banksiana* / 11-VII-2011, (1, MRNQ), *Pinus banksiana*; **Mont Saint-Hilaire**, **La Vallée-du-Richelieu**, 4-VI-2011, (1, LFRC); **Parc** **de** **la** **Mauricie**, **Le Centre**-**de**-**la**-**Mauricie**, 5-VI-2000, (1, LFRC), *Pinus strobus*; **Saint**-**Basile**, **Portneuf**, 13-VII-2011, (1, MRNQ), *Pinus banksiana*; **Sainte**-**Luce**, **La Mitis**, 1-VI-1987, (3, MRNQ), *Pinus resinosa*; **Saint**-**Élie**-**d’Orford**, **Sherbrooke**, 22-VIII-1978, (3, MRNQ), *Pinus resinosa*; **Saint**-**Zéphirin**-**de**-**Courval**, **Nicolet**-**Yamaska**, 15-VI-1982, (2, MRNQ), *Pinus resinosa*; **Valcartier**, **La Jacques**- **Cartier**, 3-VIII-1981, (3, MRNQ), *Pinus resinosa*; **Lac Beemer**, **Le Domaine**-**du**-**Roy**, 18-VII-2007, (1, MRNQ), *Pinus banksiana*; **Shenley Township, Beauce**-**Sartigan**, 2-VII-1981, (1, MRNQ), *Pinus resinosa*; **Saint**-**Chrétien**, **Charlevoix**-**Est**, 31-VIII-1984, (3, MRNQ), *Pinus banksiana*; **Baie**-**Trinité**, **Manicouagan**, 10-VI-1987, (1, MRNQ), *Pinus banksiana* / 31-V-1999, (3, MRNQ), *Pinus resinosa* / 1-VI-1999, (3, MRNQ), *Pinus resinosa*; **L’Annonciation**, **Antoine**-**Labelle**, 28-VII-1988, (4, MRNQ), *Pinus resinosa*; **Saint**-**Patrice**-**de**-**Beaurivage**, **Lotbinière**, 30-VIII-1989, (6, MRNQ), *Pinus resinosa*; **Landrienne**, **Abitibi**, 15-VII-1987, (1, MRNQ), *Pinus banksiana*; **Saint**-**Dominique**, **Abitibi Ouest**, 14-VII-2011 (1, MRNQ), *Pinus banksiana*; **Lachenaie**, **Les Moulins**, 5-VI-2000, (6, LFRC); **Saint**-**Amable**, **Marguerite D’Youville**, various dates in the period 2008–2012, (12594, INRS-IAF), *Pinus strobus* / 25-V-2000, (2, MRNQ), *Pinus strobus*; **Saint**-**Claude**, **Le Val**-**Saint**-**François**, 31-VII-1981, (9, MRNQ), *Pinus sylvestris* / 31-V-1985, (18, MRNQ), *Pinus resinosa* / various dates in the period 2008-2012, (380, INRS-IAF), *Pinus strobus*; **Fort-Coulonge**, **Pontiac**, various dates in the period 2008-2012, (3391, INRS-IAF), *Pinus strobus*/ 4-VII-2007, (1, MRNQ), *Pinus* resinosa; **Lac Cayamant**, **La Vallée**-**de**-**la**-**Gatineau**, various dates in the period 2008-2012, (38, INRS-IAF), *Pinus strobus*; **Saint**-**Simon**-**les**-**Mines**, **Beauce**-**Sartigan**, various dates in the period 2008-2012, (564, INRS-IAF), *Pinus strobus*; **Cap-Tourmente**, **La Côte**-**de**-**Beaupré**, various dates in the period 2008-2012, (368, INRS-IAF), *Pinus strobus*; **Saint**-**Étienne**, **Lévis**, 2-VI-1981, (1, CCC) / 10-VI-1983, (1, CCC); **Les Escoumins**, **La Haute**-**Côte**-**Nord**, 18-VI-1984, (3, CCC).

#### Distribution in Canada.

**ON**, **QC**, **NB**, **NS**.

### Pityophthorus consimilis

LeConte, 1878

http://species-id.net/wiki/Pityophthorus_consimilis

Fig. 10

#### Records from Bright (1981).

**Sainte-Anne-de-Bellevue, Montréal**, ?, (4, CNC); **Gaspé** Co., 15-VIII-1934, (1, CNC), *Picea glauca*; **Kazabazua, La Vallée-de-la-Gatineau**, 13-VII-1967, (52, CNC), *Pinus banksiana*; **Sainte-Julienne** (**Kelly’s Camp**), Montcalm, 17- VII-1939, (2, CNC), *Picea glauca*; **Lac Mud, Papineau**, 24-X-1967, (1, CNC).

#### New records.

**Cloutier, Rouyn-Noranda**, 30-VII-1981, (1, MRNQ), *Pinus banksiana*.

#### Distribution in Canada.

**AB, BC, MB, SK, ON, QC, NS.**

### Pityophthorus murrayanae murrayanae

Blackman, 1922

http://species-id.net/wiki/Pityophthorus_murrayanae_murrayanae

Fig. 10

#### Records from Paquin and Dupérré (2001).

**Lac Duparquet, Abitibi**, 19-VI-1994, (1, LEMU); **Jamésie**, 15-VI-1997, (1, LEMU), *Picea mariana* / 29-VI-1997, (1, LEMU), *Picea mariana* / 13-VII-1997, (1, LEMU), *Picea mariana* / 27-VII-1997, (1, LEMU), *Picea mariana* / 6-VIII-1997, (1, LEMU), *Picea mariana* / 28-IX-1997, (1, LEMU), *Picea mariana*.

#### New records.

**Fort-Coulonge, Pontiac**, various dates in the period 2008-2012, (1005, INRS-IAF), *Pinus banksiana*; **Lac Cayamant, La-Vallée-de-la-Gatineau**, various dates in the period 2008-2012, (60, INRS-IAF), *Pinus banksiana*; **Lac Villebois, Jamésie**, 14-IX-2006, (1, MRNQ), *Pinus banksiana*.

#### Distribution in Canada.

**NT, AB, BC, MB, ON, QC, NB.**

**Figure 1.**
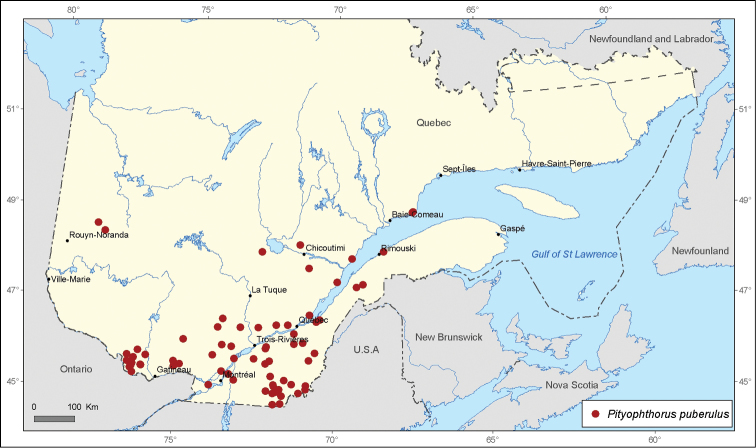
Map of *Pityophthorus puberulus* (LeConte) records in Quebec, Canada.

**Figure 2.**
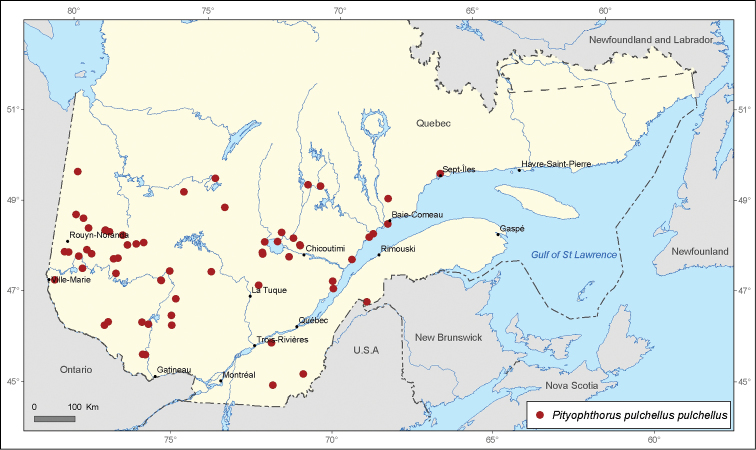
Map of *Pityophthorus pulchellus pulchellus* Eichhoff records in Quebec, Canada.

**Figure 3.**
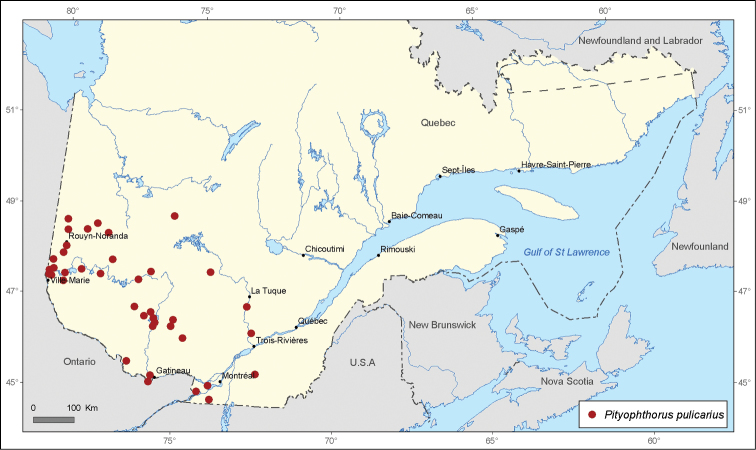
Map of *Pityophthorus pulicarius* (Zimmermann) records in Quebec, Canada.

**Figure 4.**
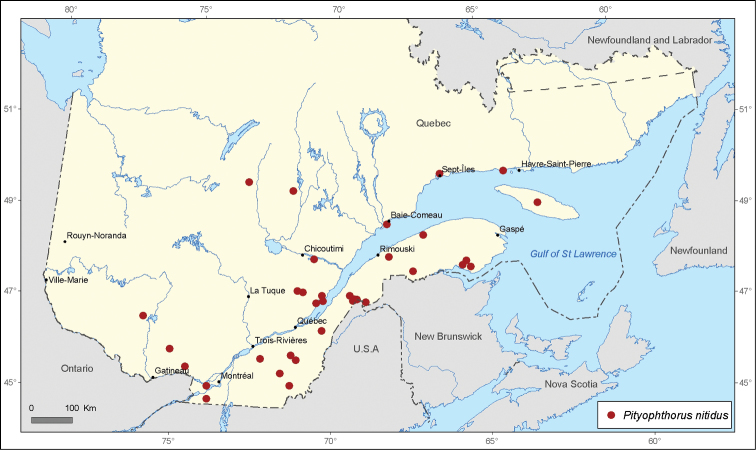
Map of *Pityophthorus nitidus* Swaine records in Quebec, Canada.

**Figure 5.**
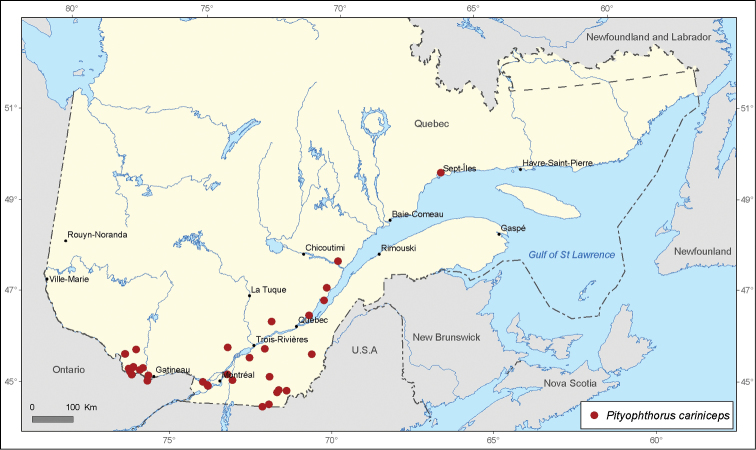
Map of *Pityophthorus cariniceps* LeConte & Horn recordsin Quebec, Canada.

**Figure 6.**
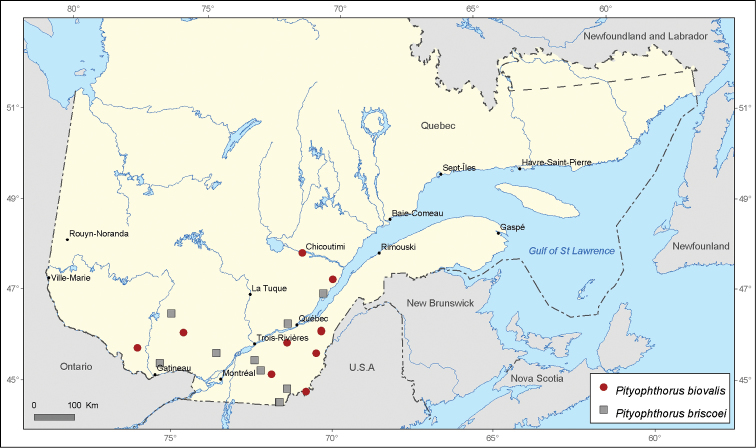
Map of *Pityophthorus biovalis* Blackman and *Pityophthorus briscoei* Blackman records in Quebec, Canada.

**Figure 7.**
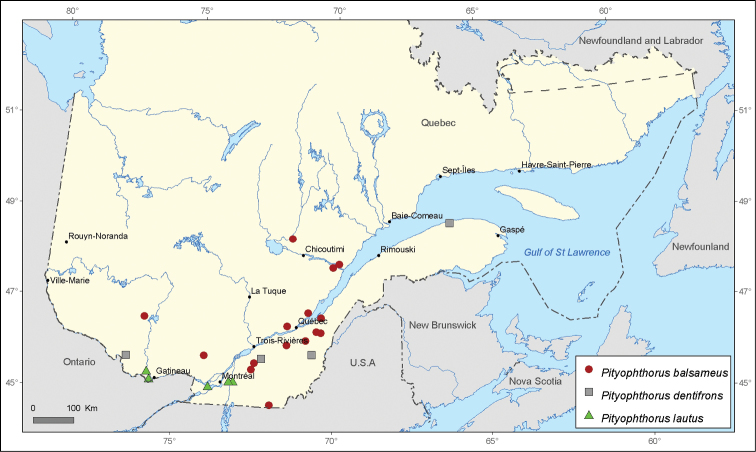
Map of *Pityophthorus balsameus* Blackman, *Pityophthorus dentifrons* Blackman and *Pityophthorus lautus* Eichhoff records in Quebec, Canada.

**Figure 8.**
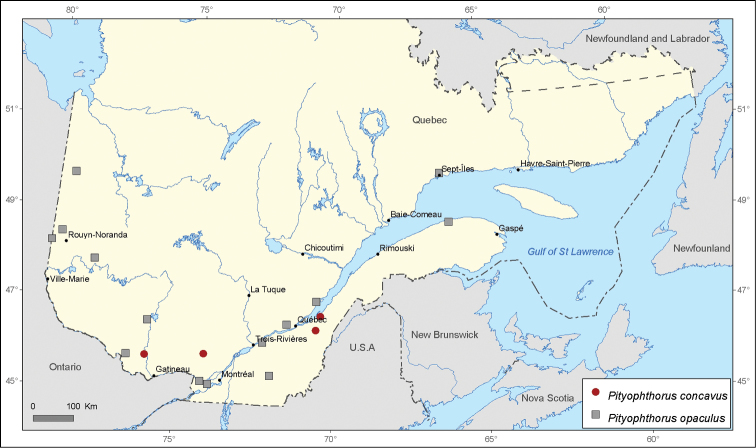
Map of *Pityophthorus concavus* Blackman and *Pityophthorus opaculus* LeConte records in Quebec, Canada.

**Figure 9.**
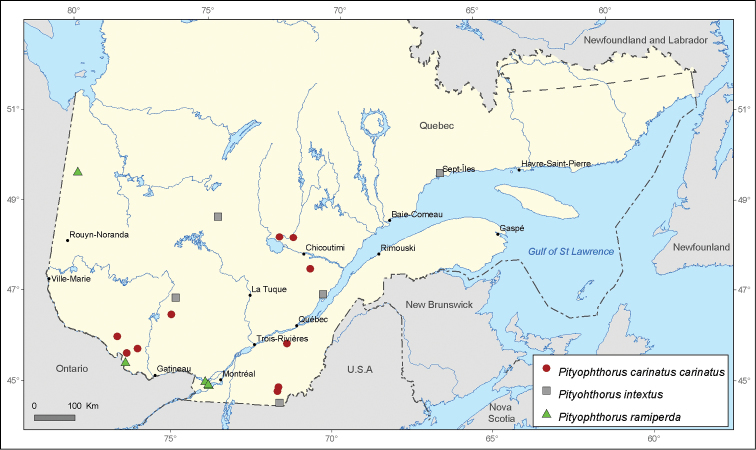
Map of *Pityophthorus carinatus carinatus* Bright, *Pityophthorus intextus* Swaine and *Pityophthorus ramiperda* Swaine records in Quebec, Canada.

**Figure 10.**
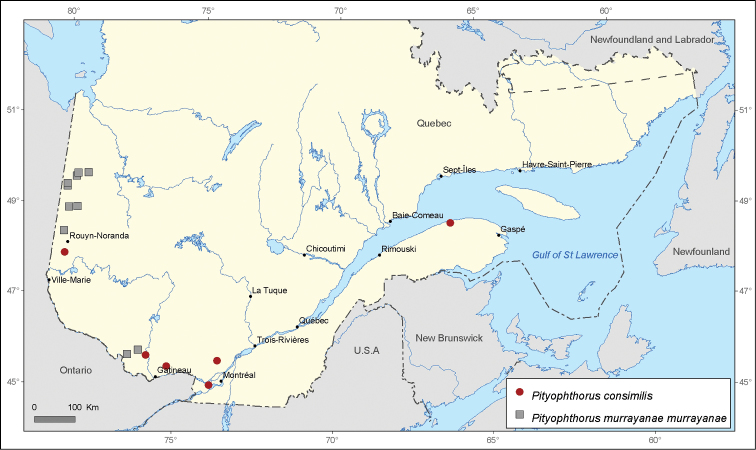
Map of *Pityophthorus consimilis* LeConte and *Pityophthorus murrayanae murrayanae* Blackman records in Quebec, Canada.

## Discussion

A total of 21 690 specimens of *Pityophthorus* originating from nine entomological collections were analyzed. A total of 291 new localities in Quebec, Canada were recorded for the 17 identified species. The most widespread species in the province of Quebec is *Pityophthorus puberulus* with 72 records, followed by *Pityophthorus pulchellus pulchellus* with 62 records, then by *P*.* pulicarius* with 40 records, *Pityophthorus nitidus* with 34 records, and *Pityophthorus cariniceps* with 33 records.

*Pityophthorus puberulus* displays a typical northeastern distribution in North America. This twig beetle breeds in various species of *Pinus*, as well as *Abies* and *Picea* (Bright 1981). All *Pityophthorus puberulus* specimens examined in the province of Quebec were found only on *Pinus* sp., particularly on *Pinus strobus* and *Pinus resinosa* and rarely on *Pinus sylvestris* and *Pinus banksiana*. Accordingly, the distribution of *Pityophthorus puberulus* in Quebec is positively correlated with the distributions of *Pinus strobus* and *Pinus resinosa*. *Pityophthorus puberulus* is also a very abundant species, and thousands of specimens may be collected during a field trapping season. Sixty-nine new distribution records are presented for this species in Quebec, Canada. According to Deyrup and Kirkendall (1983), *Pityophthorus puberulus* reproduces exclusively parthenogenetically at least in part of their distribution area, which may explain their high abundance. Nevertheless, Bright (1981) described the male of this species.

*Pityophthorus pulchellus pulchellus* is one of the most widespread species in North America (Bright 1981). The specimens of this species captured in Quebec were found on different pine species, although mainly on jack pine, *Pinus banksiana*. It is highly likely that the lack of occurrence in the northern area of Quebec may be determined by the lack of samples above 51°N. Fifty-eight new locality records are presented for this species in Quebec.

*Pityophthorus pulicarius* displays a distribution that is predominantly toward the western area of the province. This species was found in thirty-three new localities in Quebec. The specimens were collected principally on *Pinus banksiana*, as well as *Pinus strobus* and *Pinus resinosa*. *Pityophthorus pulicarius* may be an aggressive species that attacks living twigs of different species of pine (Craighead 1950).

*Pityophthorus nitidus* is a species that is widespread in North America throughout the northern coniferous forest (Bright 1981). This species breeds in different species of *Pinus* and *Picea*. In Quebec, this species is distributed predominantly along the Saint-Lawrence River through Anticosti Island, which is the easternmost record (Fig. 4). Thirty-one new records are presented for this species in Quebec.

The distribution of *Pityophthorus cariniceps* is confined to the southern and, specifically, the southeastern areas of Quebec. Two focal regions of occurrence may be detected for this species in Quebec: the first one is located in Gatineau Valley and the second stretches along the Saint-Lawrence River (Fig. 5). Twenty-nine new distribution records are provided for this species in Quebec. Bright (1981) recognized two types of morphological variation of the female frons and male declivity across the North American distribution area: “*canadensis* form”, which is more frequent within the boreal populations, and “*cariniceps* form”, which predominates in the southern populations. The specimens of *Pityophthorus cariniceps* analyzed from the province of Quebec displayed both previously mentioned forms and an extreme variation of the “*cariniceps* form” with a strong and sharp elevation of the female frons. *Pityophthorus cariniceps*, *Pityophthorus biovalis*, *Pityophthorus carinatus carinatus*, *Pityophthorus balsameus*, *Pityophthorus briscoei*, and *Pityophthorus concavus* compose the “cariniceps group”. All of these species within the “cariniceps group” are identified mainly according to characteristics of the female frons. However, a significant morphological variation of female frons could be detected in each of the species belonging to this taxonomic group. These variations are sometimes so large that they may lead to misidentification. Therefore, a taxonomic revision of the “cariniceps group” through a combination of their morphological and molecular traits is required.

Several species of *Pityophthorus* occurring in Quebec, Canada display a narrow distribution across the territory. These include *Pityophthorus consimilis* with 6 records, *Pityophthorus ramiperda* and *Pityophthorus intextus* with 5 records, and *Pityophthorus dentifrons* and *Pityophthorus concavus* with only 4 records each.

*Pityophthorus consimilis* is a rare species in Quebec. Only one new provincial record is presented in the western part of Quebec. The species is also rare in the province of Nova Scotia with only one record (Majka et al. 2007a) and it is absent in other Maritime provinces of Canada (Majka et al. 2007a; Majka et al. 2007b).

*Pityophthorus ramiperda* is a very rare species with a limited distribution in the eastern part of North America. This species was initially reported in Canada only in Ontario and Quebec by Bright (1981) and was relatively recently found in the province of Nova Scotia (Majka et al. 2007a). Three new locality records are provided in the province of Quebec for this species, which was most recently cited by Paquin and Dupérré (2001) in the Jamésie Regional County Municipality. This record is the northernmost distribution point of *Pityophthorus ramiperda* in North America (Fig. 9). This specieswas previously captured only on white pine, *Pinus strobus*. However, in the northern part of Quebec territory, the species has been captured in a *Picea mariana* stand far beyond the northern distribution limit of *Pinus strobus* in the province. Consequently, it is highly likely that *Pityophthorus ramiperda* breed in other coniferous species and not just in white pine. This hypothesis remains to be confirmed.

*Pityophthorus intextus* was first mentioned in Quebec by Laplante et al. (1991) and later in a publication of the Ministère des Ressources Naturelles du Québec (MRNQ 2008), but no locality records were provided. In the taxonomic monograph published by Bright (1981), this species was not mentioned as present in Quebec. We present five new localities for *Pityophthorus intextus* in Quebec, and these provide the first reliable distribution data for this species. A closely related species, *Pityophthorus cascoensis*, which is known to be found in the Northwest Territories, Alberta, Ontario, Newfoundland and Labrador has never been reported in Quebec (Bright 1981; Bright and Skidmore 1997, 2002; Wood and Bright 1987, 1992). According to the distribution in Canada, this species should also be found in Quebec.

*Pityophthorus dentifrons* displays a predominantly southeastern distribution in North America. It is also a species with scarce representation in Quebec. Only three new locality records are provided.

*Pityophthorus concavus* has an eastern North American distribution, as reported by Bright (1981). Although Wood and Bright (1992) mention some records from British Columbia, the data do not appear to be reliable because no specimen originating from this Canadian province is inventoried in CNC. This species is rare in Quebec. Three new records are included in this article, and these constitute the only records of this species since its first mention in the province. No other specimen has been recaptured recently.

The distributions of *Pityophthorus lautus*, *Pityophthorus biovalis*, and *Pityophthorus murrayanae murrayanae* display some peculiarities:

*Pityophthorus lautus* is a species found in mixed and deciduous forests in Quebec and displays a typical southern distribution. The reduced number of locality records in Quebec may be somewhat explained by the placement of the sampling stations, which were located predominantly in coniferous stands. Consequently, the distribution of this species that we report across the province could be underestimated.

*Pityophthorus biovalis*, similarly to *Pityophthorus intextus*, was mentioned by Laplante et al. (1991) and in the publication of Ministère des Ressources Naturelles du Québec (MRNQ 2008), but no locality records are provided. Thus, we consider all nine locality records as new for the province of Quebec.

*Pityophthorus murrayanae murrayanae* provided a very interesting case.This subspecies was first recorded in Quebec by Paquin and Dupérré (2001) in the Jamésie Regional County Municipality, which is also the northernmost mentioned distribution record in Quebec. Three new locality records are provided in this paper for *Pityophthorus murrayanae murrayanae*, and these represent the second report of this subspecies in Quebec. According to its actual provincial distribution, *Pityophthorus murrayanae murrayanae* is found only in the northwestern area of Quebec (Fig. 10). Ten specimens from the CNC collection belonging to this subspecies were verified for morphological variation. Specimens from Alberta (3 specimens), British Columbia (3 specimens), Ontario (2 specimens), and New Brunswick (2 specimens) were analyzed. We observed a slight variation in the dimensions of the granules of the elytra declivity. The specimens with west Canadian occurrence display larger granules, whereas the specimens originating from New Brunswick harbor very small granules. The size of the granules on the declivity may also vary among sexes. Another variable morphological character in *Pityophthorus murrayanae murrayanae* is related to the pubescence of the female frons (Bright 1981). However, this morphological variation occurs within a population (location) and not necessarily between different locations within the full distribution of the species.

The field samples gathered between 2008 and 2012 in the province of Quebec allowed us to identify some interesting specimens (three specimens) that could not be assigned to any known *Pityophthorus* species. Despite our increased sampling effort, no other similar specimens were recaptured. Further studies will be needed to confirm if novel *Pityophthorus* species could be described or these are simply morphological anomalies.

## Conclusions

1. More than 30 % (17 species) of the *Pityophthorus* fauna recorded in Canada is found in Province of Quebec.

2. In general, the our reported distributions of all *Pityophthorus* species in Quebec may be biased by the locations of our permanent sampling stations, which were positioned exclusively in conifer seed orchards and predominantly along the primary river valleys and major roads. The greatest number of distribution points is concentrated along the Saint-Lawrence River and Gatineau Valley. The actual positions of the permanent sampling stations in Quebec are principally connected with the timber industry. Future sampling campaigns should be organized above 51°N to obtain a more realistic overview of the distribution of *Pityophthorus* in Quebec.

3. Diverse types of forest ecosystems, as well as the north-south temperature gradient may potentially shelter more than 17 *Pityophthorus* species. Further studies will be needed to increase the knowledge on the fauna and taxonomy of this twig beetle group in Quebec, Canada.

## Supplementary Material

XML Treatment for Pityophthorus lautus

XML Treatment for Pityophthorus pulicarius

XML Treatment for Pityophthorus nitidus

XML Treatment for Pityophthorus intextus

XML Treatment for Pityophthorus pulchellus pulchellus

XML Treatment for Pityophthorus cariniceps

XML Treatment for Pityophthorus biovalis

XML Treatment for Pityophthorus carinatus carinatus

XML Treatment for Pityophthorus balsameus

XML Treatment for Pityophthorus briscoei

XML Treatment for Pityophthorus concavus

XML Treatment for Pityophthorus ramiperda

XML Treatment for Pityophthorus opaculus

XML Treatment for Pityophthorus dentifrons

XML Treatment for Pityophthorus puberulus

XML Treatment for Pityophthorus consimilis

XML Treatment for Pityophthorus murrayanae murrayanae
